# Acknowledgement to Reviewers of *Insects* in 2017

**DOI:** 10.3390/insects9010006

**Published:** 2018-01-11

**Authors:** Insects Editorial Office

**Affiliations:** MDPI AG, St. Alban-Anlage 66, 4052 Basel, Switzerland

Peer review is an essential part in the publication process, ensuring that *Insects* maintains high quality standards for its published papers. In 2017, a total of 127 papers were published in the journal. Thanks to the cooperation of our reviewers, the median time to first decision was 24 days and the median time to publication was 62 days. The editors would like to express their sincere gratitude to the following reviewers for their time and dedication in 2017:

Aak, AndersLövei, Gabor L.Abro, G. H.Lower, Sarah E.Addesso, KarlaLuchetti, AndreaAdler, PeterLuvall, Jeffrey C.Agerbirk, NielsMackay, William P.Agnello, ArthurMadeira, Filipe NogueiraAkhtar, YasminMaisonhaute, Julie-ÉléonoreAllan, SandraMalacrinò, AntoninoAlyokhin, AndreiMartínez, Luis CarlosAmendt, JensMarzachi, CristinaAmiri, EsmaeilMassimino Cocuzza, GiusepeArango, Rachel A.Maxwell, Michael R.Arrieta, Juanita RodriguezMcfadyen, Rachel E.c.Asgari, SassanMebs, DietrichAubret, FabienMedrzycki, PiotrBagnoli, BrunoMehlhorn, HeinzBanks, John EdwardMerritt, DavidBanschbach, Valerie S.Merritt, Richard W.Barclay, MaxMikonranta, LauriBarman, ApurbaMillar, JocelynBasche, AndreaMiller, JimBažok, RenataMilotic, TanjaBecher, Paul G.Mody, KarstenBenecke, MarkMogren, ChristinaBeresford, DavidMohr, Rachel M.Beugnet, FredericMoore, HannahBeza, CristianMorales-Ramos, Juan A.Bixler, Robert D.Moreau, GaétanBlatt, SuzanneMori, BoydBokhorst, StefMorrell, JeffBooth, WarrenMorrison, Lloyd W.Borges, Paulo A. V.Morrison, William R.Bose, JoyMottern, Jason L.Bourgeois, LanieMullin, ChrisBradshaw, TerenceMuniappan, Rangaswamy(Muni)Bradshaw, TerenceMurchie, Archie K.Brénière, Simone FrédériqueMussury, Rosilda M.Brett, BlaauwNanetti, AntonioBrown, William D.Nehring, VolkerBrundage, AdrienneNice, ChrisBryant, BartNino, ElinaBüchs, WolfgangNishiwaki, HisashiBurks, CharlesO’neill, KevinBurritt, James BObrycki, JohnCammack, JonathanOgórek, RafałCastle, StevenOi, FaithCaterino, MichaelOkabe, KimikoChang, Chiou LingOppenheim, SaraCharabidze, DamienOsada, YoheiCharrel, RémiOsbourn, AnneCheng, XiaoyanPappas, Maria L.Choe, Dong-HwanPark, Kye ChungChristiaens, OlivierPatt, JosephCornelius, MaryPatten, KimCrozatier-Borde, MichelePechal, JenniferCurrie, Cameron R.Peletto, SimoneCuthbertson, Andrew G. S.Perring, Thomas M.Daane, Kent. MPetrakis, Panos. V.Dadour, Ian R.Pfeiffer, DouglasDahlem, Gregory A.Phillips, ThomasDe Souza, Danival JoséPicard, ChristineDeBano, SandyPlarre, RuedigerDenux, OlivierPleasants, John MDi Prisco, GennaroPleydell, DavidDitrich, TomášPolaszek, AndrewDoggett, StephenPondeville, EmilieDohmen-Vereijssen, JessicaPons, XavierDooremalen, Coby VanPoyet, MathildeDorin, AlanPozsgai, GaborDoublet, VincentPozzebon, AlbertoDrijfhout, FalkoPrischmann-Voldseth, DeirdreDufour, Bernard P.Ramasamy, SrinivasanDurdle, AnnalisaReimer, Adam P.Duso, CarloReisen, William K.Dutcher, James D.Renkema, Justin M.Dyer, AdrianResh, VincentEdwards, Marten JohnRiehle, MichaelEger, JosephRing, DennisEizaguirre, MatildeRinkevich, FrankEllsworth, Peter C.Rivera-Pérez, CrisalejandraEndersby, N.M.Roe, AmandaErler, SilvioRojo, SantosEvans, BRADEN G.Römer, DanielaEvenden, Maja L.Romero, AlvaroEwing, CurtisRondoni, GabrielleFellowes, MarkRossi-Stacconi, Marco ValerioFernández, Irene GarcíaRuberson, John R.Finke, Mark D.Rumble, HeatherFleming, DanielRust, MichaelFowler, SimonRybska, ElizaFranca, Filipe MachadoSangha, Jatinder SinghFrank, Daniel L.Saunders, ManuGangloff-Kaufman, JodyScharf, InonGanjisaffar, FatemehScharf, MichaelGarrick, RyanScheffrahn, Rudolf H.Gezon, Zachariah J.Schetelig, Marc F.Giorgini, MassimoScott, IanGippet, Jérôme M. W.Seeley, ThomasGnanvossou, DesireSeverns, Paul M.Gogol-Döring, AndreasSgolastra, FabioGols, RietaShearer, Peter W.Gondhalekar, Ameya D.Shelomi, MatanGrab, HeatherShelton, ThomasGrabenweger, GiselherShik, Jonathan Z.Gradish, A. E.Shrestha, SonyGreen, FrederickShuey, JohnGroßhans, JörgSial, Ashfaq AhmadGruner, SusanSmartt, Chelsea T.Guastella, DevidSmith, Hugh A.Guerrero, AngelSmith, JudithGut, LarrySmodiš Škerl, MajaGuzmán-Novoa, ErnestoSolensky, MichelleHabel, Jan ChristianSolis, AlmaHall, DavidSommaggio, DanieleHarbison, JustinSpies, JanineHart, AdamStamper, TrevorHatt, SéverinStark, John D.Hayford, BarbaraSteckel, JulianeHeadrick, David H.Stefano, CassanelliHealy, KristenStelinski, LucaszHeerman, MatthewStevenson, Philip C.Helantera, HeikkiStock, PatriciaHelden, Maarten VanStockan, JenniHenderson, GreggStökl, JohannesHendriksma, HarmenSuiter, DanHenry, MorganeSylvain, PoggiHenry, Thomas J.Tabata, JunHimler, AnnaTamburini, GiovanniHoffmann, Benjamin D.Tarone, AaronHoffmann, KlausTaylor, DavidHogsette, Jerome A.Thomassin, PaulHolmes, MichaelThomson, LindaHopkinson, JamieTillman, Patricia G.Horowitz, A. RamiTixier, Marie-StéphaneHorton, David RToft, SørenHottel, BenjaminTomažič, IztokHowell, KateTrager, James C.Hu, David L.Trdan, StanislavHughes, GabrielTrematerra, PasqualeHvenegaard, Glen T.Tronstad, RussellIbrahim, Sulaiman SadiTrumbo, StephenIngram, ErinTsagkarakou, AnastasiaIsman, MurrayTschinkel, Walter R.Jandt, JenniferTseng, Shu-PingJaronski, StefanTuda, MidoriJavan, GulnazTurroni, SilviaJenkins, NinaUnlu, IsikJohnson, D. T.Van Den Hurk, AndrewJohnston, J. SpencerVan Der Borght, MikJones, CarolVan Der Werf, WopkeJones, IanVan Leeuwen, ThomasJordan, HeatherVanlandingham, DanaJudd, TimothyVarloud, MarieKaiser, Matthew C.Vilcinskas, AndreasKamble, ShripatVogelweith, FannyKampen, HelgeVoss, Sasha C.Karimzadeh, RoghaiyehVosteen, IlkaKim, Ke ChungVuts, JozsefKlassen, Jonathan LWagner, SvenKlotz, Stephen A.Walgenbach, JamesKnight, KathleenWaller, DeborahKnyshov, AlexanderWallner, Adam MKobori, Y.Wang, CaiKolev, YankoWatanabe, MamoruKooij, Pepijn W.Watson, Gillian W.Kozlov, Mikhail V.Webster, BenKrams, IndrikisWeeks, EmmaKristensen, MichaelWeeks, FaithKryger, PerWeetman, DavidKyle, ChristopherWells, Carrie N.Lahiri, SriyankaWells, JeffreyLake, EllenWenninger, ErikLandolt, Peter J.Wetterer, James K.Langrock, RolandWilson, HoustonLarsen, Diane L.Wise, JohnLeather, SimonWolf, StephanLeBlanc, HeleneWood, ThomasLeBrun, EdwardWoodring, JosephLecocq, AntoineWu-Smart, JudyLee, Jana C.Wybouw, NickyLees, RosemaryYanagawa, AyaLeibovici, DidierYang, Chin-ChengLents, NathanYang, Chiou-HerrLeonhardt, SaraYang, ShengLesnik, JulieYee, Wee L.Liberti, JoanitoYorzinski, JessicaLong, Elizabeth Y.Young, Daniel K.Loni, AugustoZhang, Qing-heLövei, GaborZhang, Yangzhuo

